# Distant metastases of a squamous cell carcinoma of the tongue in peripheral skeletal muscles and adjacent soft tissues

**DOI:** 10.1186/1746-160X-4-7

**Published:** 2008-03-26

**Authors:** Ralf Smeets, Maurice B Grosjean, Max Heiland, Dieter Riediger, Oliver Maciejewski

**Affiliations:** 1 University Hospital Aachen, Department of Oral and Maxillofacial Surgery, Aachen, Germany; 2 RWTH Aachen University, Interdisciplinary Center for Clinical Research (IZKF) 'BIOMAT', Aachen, Germany; 3 University Hospital Basel, Department of Reconstructive Surgery, Division of Cranio-Maxillofacial Surgery, Basel, Switzerland; 4 University of Basel, Center of Multidisciplinary Research in Cranio-Maxillofacial Surgery "Hightech-Forschungs-Zentrum" (HFZ), Basel, Switzerland; 5 University Hospital Hamburg-Eppendorf, Department of Oral and Maxillofacial Surgery, Hamburg, Germany

## Abstract

A 66-year-old female patient was admitted to our department with a large tumor of the tongue measuring 10 cm in diameter. The tumor occupied nearly the entire oral cavity and showed exophytic and ulcerative areas. Histological analysis revealed a low grade squamous cell carcinoma (SCC) of the tongue. Bilateral enlarged cervical lymphatic masses were also present. The extent of the tumor infiltration was assessed by fluoro-2-deoxy-glucose-positron emission tomography (PET) scans showing an elevated activity of the tracer corresponding to the assumed cervical metastases. Additionally, pulmonary metastases were identified. Contrast enhanced computed tomography (CT) scans showed metastases in the soft tissues of the abdomen, legs and arms. Foci of distant metastases were found in the left upper anterior thoracal wall, near the intraabdominal portion of the aorta, near the right iliac crest and in both the right vastus medialis- and adductor magnus muscles. The final diagnosis was a T4N3M1(G3)(C3) SCC of the tongue with multiple distant thoracal, abdominal and intramuscular metastases. The survival expectancy was five weeks, and the patient finally deceased by cardiopulmonary complications.

## Background

Patients suffering from a squamous cell carcinoma (SCC) of the head and neck region with distant metastases generally have a very poor prognosis [1]. Distant metastases typically manifest themselves in the lung, bones, liver and skin. Only few articles have been reported on a different localization of distant metastases. Moriya et al. (2004) recently reported on a patient suffering from a cardial metastasis of an oral SSC together with additional metastases in the liver, lung, spleen and kidneys [2]. A distant metastasis in the gluteal muscle of a 65-year-old patient suffering from a SSC of the larynx has been recently described [3]. Oo et al. (2004) have identified three patients with metastases in the axillary lymph nodes over a period of 20 years [4]. In these three case reports, the primary malignoma were identified as carcinomas within a pleomorphous adenoma of the parotideal gland, a SCC of the tongue and a SCC of the anterior floor of the mouth, respectively. Mess et al. (1986) have reported on distant metastases which were localized in the carpal bones of the midhand and in the bones of the foot [5]. As to our knowledge, no case has been reported up to date on the manifestation of distant metastases of a SCC in the soft tissue of the extremities.

In summary, we report on a 66-year-old female patient who suffered from a terminal SCC of the tongue with multiple distant metastases which were localized mainly in the peripheral skeletal muscles and adjacent soft tissues of the lower extremities. Furthermore, we show an overview on the current literature on metastases originating from head and neck tumors [Table 1].

**Table 1:** Survey of the literature

Authors	Cases	Results (localization of distant metastases in %)
Probert et al. 1974 [24]	96 Patients with SCC, 31% OSCC	lung 65%, bone 25%, liver 24%, skin 14%, brain 13%, adrenal 8%, heart 7%, kidney 6%, peritoneum, mediastinum and soft tissue each 5%, esophagus 4%, spleen 3%, bone marrow 3%, thyroid 2%, prostate 1% and middle ear 1%.
Merino et al. 1977 [25]	546 patients with SCC, 21% OSCC	primary tumor orally or in the oropharynx: lung 52%, bone 20.3%, liver 6%, mediastinum 2.9%, lung and bone 3.3% and others 15.4%. Primary tumor in the nasopharynx: bone 54%, lung 23.8%; primary tumors of the fossa tonsillaris and of the basis of the tongue: metastases were primarily found in the liver (22% and 10.8% respectiveliy)
Papac et al. 1984 [26]	52 patients with SCC, 4% at the bottom of the oral cavity, 10% tumors of the tongue	lung 75%, bone 44%, liver 17%, skin 13%, brain 13%, adrenal 6%, heart 8%, kidney 10%, GIT 15%, mediastinum 10%, spleen 3% and thyroid 6%.
Troell et al. 1995 [27]	79 patients with SCC with a total of 145 remote metastases.	lung 45, bone 27, liver 11, mediastinum 10 and other localisations (adrenal, brain, pericard, kidney and thyroid) 7.
De Bree et al. 2000 [28]	17 patients with SCC, 34% OSCC	lung 71%, mediastinum 24%, bone 24% and liver 6%.
Leon et al. 2000 [29]	64 patients with SCC, 2% OSCC	lung/mediastinum 52%, bone 12%, liver 5%, a combination of lung with bone and liver or skin 31%.
Kowalski et al. 2005 [30]	89 patients with distant metastases coming from oral or oropharyngeal SCC.	lung 58.4%, bone 37.1%, liver 3.4%, brain 3.4%, soft tissue 2.2%, peritoneum 1.1%, mediastinum 1.1%, axillary lymph nodes 1.1%, lung combined with bone 5,6%, lung combined with liver 1.1% and lung combined with brain 1.1%.
Alvarez Marcos et al. 2006 [31]	39 patients with SCC, 26% OSCC	lung 58%, bone 22%, liver 9%, soft tissue 9% and others 2%.

Abbreviations: SCC = squamous cell carcinoma.

## Case presentation

**Figure 1:**
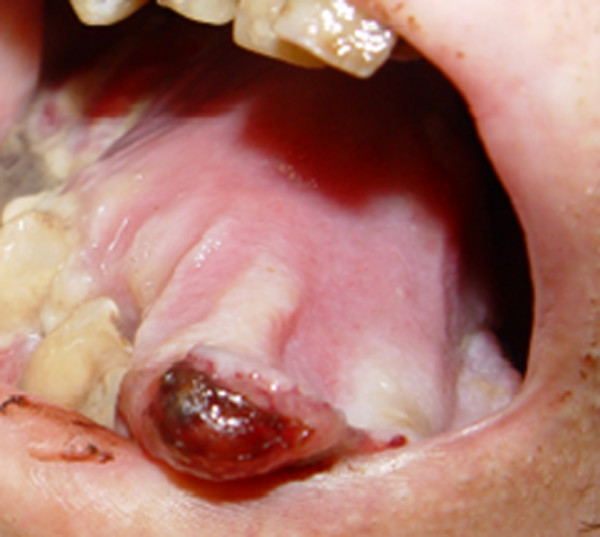
Intraoral photography: Tumor of the tongue with bite marks at the margin, filling the oral cavity.

**Figure 2:**
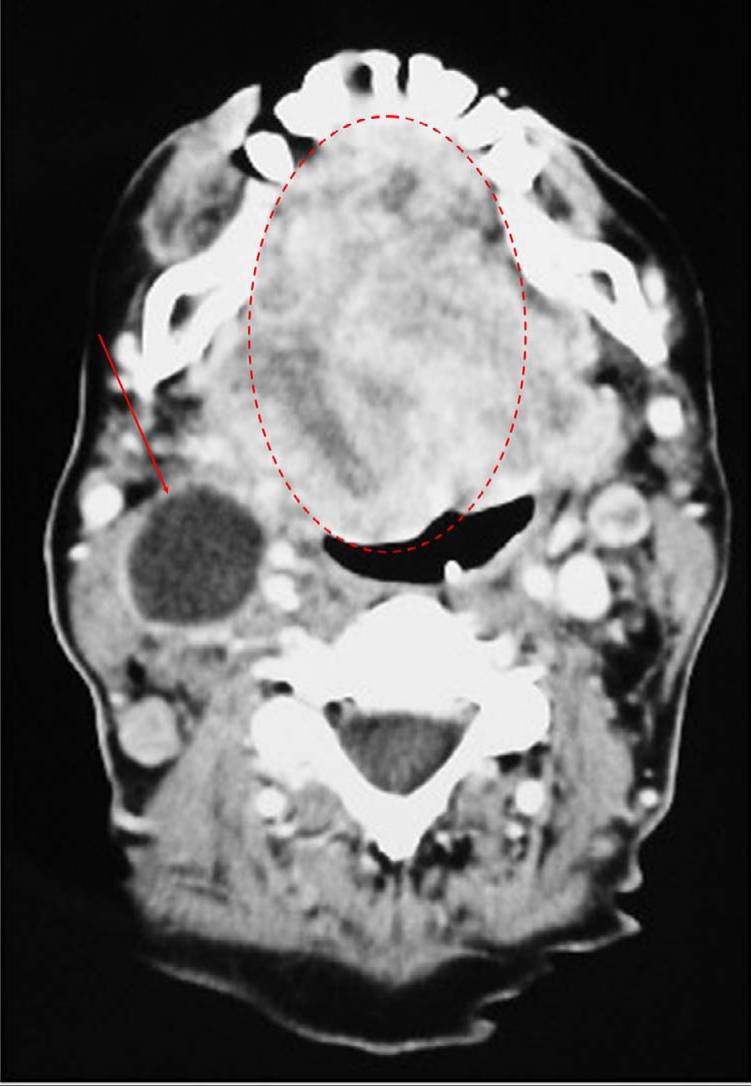
Contrast enhanced CT, soft tissue window: Tumor of the tongue (10 cm) with a large lymphe node metastasis near the right cervical vessel-nerve-sheath (red arrow).

**Figure 3:**
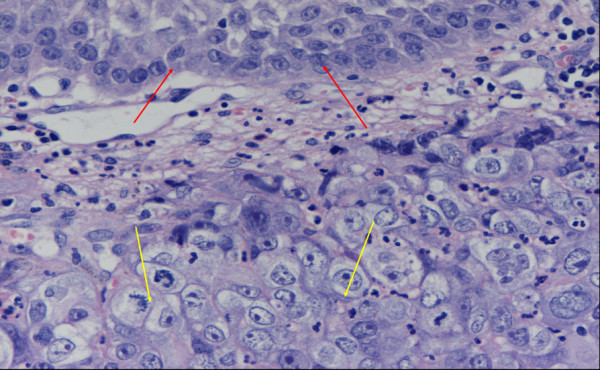
Histology: normal squamous epithelium (red arrows); carcinoma showing immature cells without keratosis which corresponds to a G3-grading (hematoxylin and eosin (HE) staining; magnification: 100×).

**Figure 4:**
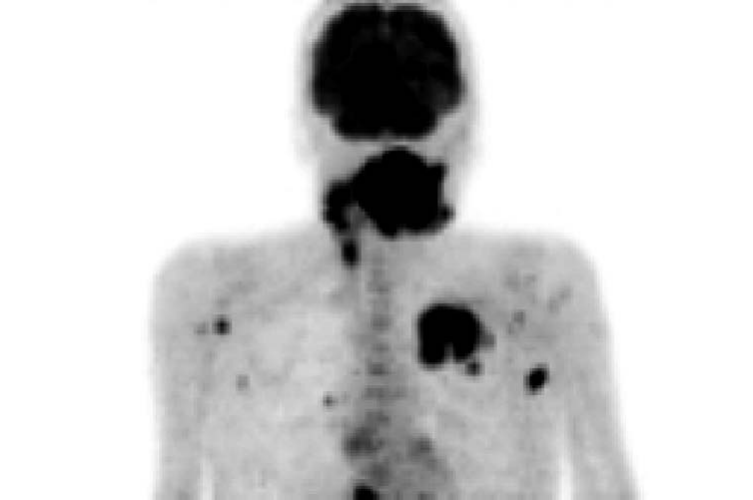
Imaging by fluorine 18-fluoro-2-deoxy-glucose-positron emission tomography (18F-FDG-PET) anterior-posterior view.

**Figure 5:**
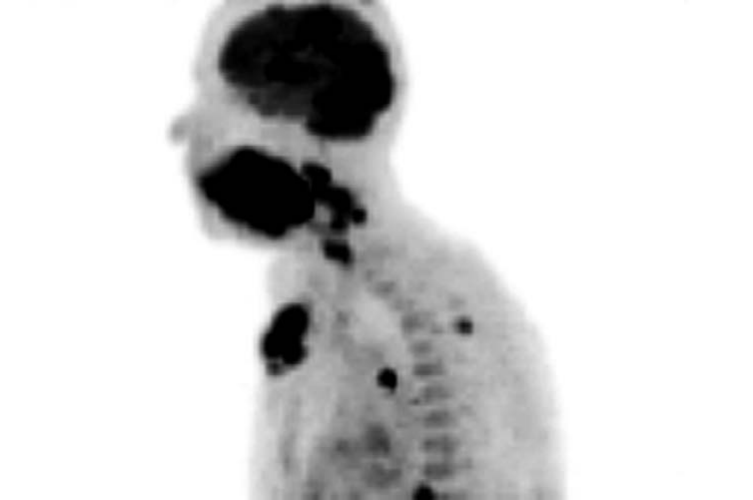
Imaging by fluorine 18-fluoro-2-deoxy-glucose-positron emission tomography (18F-FDG-PET) lateral view.

A 66-year-old woman was admitted with the symptoms of acute dyspnoe, orthopnoe and progressive dysphagia. The inspection of the oral cavity revealed an extensive lesion of the tongue (Fig. 1) with areas of exophytic growth of the tumor as well as a large ulcus located at the dorsal part of the tongue. This large tumor of the tongue occupied nearly the whole oral cavity and the anterior part of the tongue's margin clearly showed biting traces. These inspectatory findings were accompanied by a respective halitosis. Both the general and nutritional status of the patient were clearly reduced with a body mass index (BMI) of 15.6 kg/m2 (body weight of 40 kg and height of 1.60 m). The medical examination revealed the presence of bilateral large cervical conglomerates of lymph nodes that sonographically measured 6 cm in diameter. An acute obstruction of the upper airway by the tumor mass could be excluded by native computed tomography (CT) imaging of the neck and therefore a tracheotomy was not performed. At admission, the diameter of the tumor was already greater than 10 cm (Fig. 2). At the same time, a bioptic tissue probe was analyzed histologically and revealed a low grade differenciated (G3) SCC (Fig. 3). Imaging by fluorine 18-fluoro-2-deoxy-glucose-positron emission tomography (18F-FDG-PET; Fig. 4, 5) scans showed cervical hypermetabolic foci along the nerve-vessel-sheaths of both sides that were suspect for malignancy and assessed as metastases with regard to sensitivity and specifity of PET and CT scan. Multiple metastatic lesions were also present in the lung. Furthermore, foci that were suspect for metastases were also found unilaterally in the muscular system of both the left part of the back and left anterior thoracic wall. On the basis of these rather atypical findings, we performed a total body CT in order to exclude the simultaneous presence of a second malignoma.

**Figure 6:**
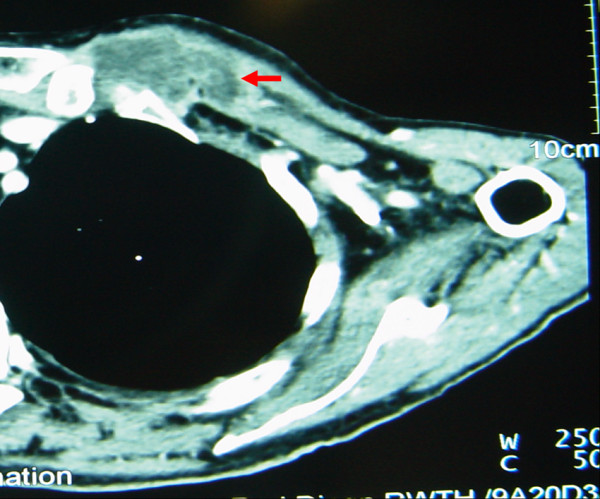
Contrast enhanced CT, soft tissue window: Soft tissue metastasis at the left upper ventral throracic wall.

**Figure 7:**
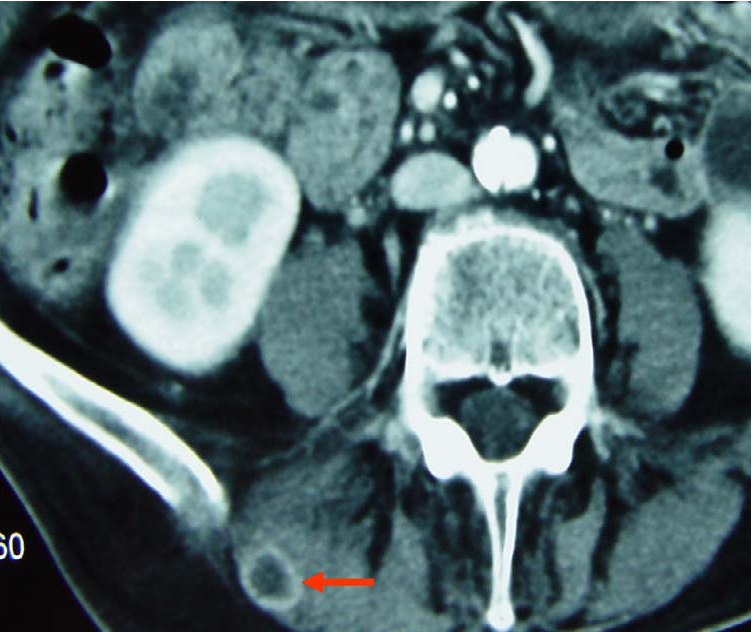
Contrast enhanced CT, soft tissue window: Soft tissue metastasis (1 cm) in the iliocostal lumborum muscle on the right side.

**Figure 8:**
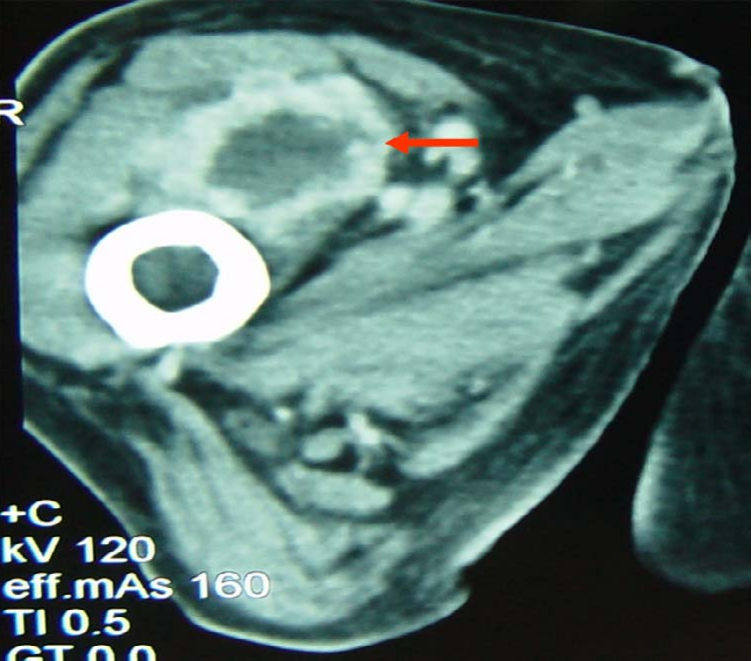
Contrast enhanced CT, soft tissue window: Soft tissue metastasis in the right vastus medialis/intermedius muscles.

Altogether, one suspect soft tissue metastasis could be identified near the left upper ventral thoracal wall (Fig. 6). A further and smaller soft tissue metastasis was found caudal of the sterno-costal base of the first rib in close vicinity to the internal mammary artery. Another metastasis emerged within the thoracic cavity in the right paraaortal soft tissue sheath. A metastasis measuring approximately 1 cm in diameter was detected in the right iliocostal lumborum muscle near the pelvic rim (Fig. 7). In the right vastus medialis/intermedius muscle one soft tissue metastasis of a diameter of several centimeters was found (Fig. 8). Also, more caudal, a metastasis of approx. 1 cm in diameter was shown in the right adductor magnus muscle. In the long head of the left biceps femoris muscle another metastasis measuring a few millimeters was identified.

In summary, the tumor staging according to the UICC (Union internationale contre le cancer) revealed a terminal cT4cN3cM1(G3)(C3) tumor. At this advanced stage, surgical therapy with curative intention was not indicated. At the same time, the patient strictly refused a surgical reduction of the tumor, a palliative radiochemotherapy as well as the application of a PEG-device. Therefore, she was transferred to the palliative care unit. The patient finally deceased after 5 weeks of hospitalization by a cardiopulmonary failure as a consequence of the tumor progression. The histologic confirmation of the suspect radiologic diagnoses, which included the spreading of the neoplasia, could be made only for the intraoral findings within the limits of the primary diagnostics, because the patient's relatives did not consent to perform an autopsy despite our request.

## Conclusion

Importantly, in the case presented here, the pattern of an advanced hematogenous spreading of metastases is particular. The metastases were located mainly in the soft tissues of the lower extremities, in particular in the skeletal muscular system. A few small metastasis-like lesions were also found in the cardiopulmonary system, as shown by 18F-FDG-PET imaging and CT scans, but they were clearly of minor extent if compared to the prominent distant metastases in the peripheral skeletal muscles and adjacent soft tissues.

The patient did not show any cardiac lesion in the CT scans. Thus, in case of the presence of masses of unknown origin in the lower extremities in patients who once suffered or are still suffering from a SCC of the oral cavity, a possible spreading of metastases should be taken into consideration. We would like to point out that the examination for such findings should be performed during the continuous tumor recall and control appointments. In the case presented here, a native computertomography of the neck was initially performed. It showed no obstruction or imminent obstruction of the airways. Therefore a tracheotomy was abstained from. In general and in accordance with the current literature, the authors prefer a preoperative tracheotomy in order to avoid postoperative airway difficulties and a tracheotomy in an emergeny setting [6].

In the case of the patient presented here, the quality of life was limited mainly by the tumor of the tongue causing impaired ability to swallow, reduced function of speech and impairment by esthetic and functional disfigurement, three factors that contributed to a low quality of life in a considerable degree. Not the distant metastases were the life-limiting factor, but rather the extensive local findings in the oral cavity which caused considerable respiratory and nutritional limitations. A valuable alternative for patients with a stage IV tumor disease with distant metastases, where the primary tumor is mostly considered to be the survival-limiting factor, is to offer them both an immediate therapy of the primary tumor and an integral palliative care in order to improve the quality of life. Other authors also support these recommendations; e.g., it has recently been shown that patients whose loco-regional and remote metastases were operated had a high two-year surviving rate [7]. This kind of tumor-debulking operations cause important defects in the orofacial area and should often be covered by microsurgical flaps. If the patient shows a rather reduced general state of health, any tumor-debulking operation should probably be excerted in a two-timed planning concept to minimize the operative risk. Other authors have recently reported that immediate microsurgical reconstructions could be beneficial in palliative situations [8,9].

It has recently been reported that some patients who suffered an untreated SCC of the head and neck region survived for more than five years. Studies in which the outcome of patients with a SCC who were not treated at all were compared to the outcome of patients who underwent a palliative therapy showed that the mean survival rate was 8.4 months larger in patients who underwent a palliative therapy [10]. However, other authors reported on prolonged surviving rates in some cases of untreated tumors of the head and neck [11-14]. Kowalski and Carvalho (2000, 2001) analyzed in a long-lasting retrospective study from 1953 to 1990 the clinical outcome of 808 patients with an untreated tumor of the head and neck [15,16]. They reported that, in accordance with the above findings, patients, who did not receive any treatment, survived up to 4 years [16]. Therefore, it seems to be difficult to anticipate the rate of survival by using subjective or objective criteria. The author reports that nearly 50% of patients with an untreated tumor in the head and neck region deceased within the first four month after the diagnosis had been made and given to the patient. Surprisingly, some patients survived more than four years and the author reported that the factors which had a significant impact on survival were localization of the tumor, tumor staging and scoring the performance of the patients.

Rapoport et al. (1975) compared recurring head and neck tumors in relation to the outcome [17]. One group underwent chemotherapy whereas the control group received a placebo treatment. They concluded that chemotherapy considerably improved the quality of life, but did not prolong the time of survival. New therapeutic strategies such as treatment of head and neck tumors with hyperfractional radiotherapy followed by chemotherapy showed five-year survival rates of more than 40% [18-20]. In consideration of these survival rates, Kowalski et Carvalho (2001) suggested that new treatment approaches should be offered to all patients and should start as early as possible after having given the diagnosis to the patient. This is important because the mean time period which a transition from a terminal to an untreatable tumor takes is considered to be only 3.8 months [16].

The studies cited here support the ruling opinion that a meticulous staging is an essential aspect within the control of survival rates and quality of life. If the patient, as in the case presented here, refuses biopsy of suspected peripheral metastases, further costs can evolve later when other apparative examination means become necessary. This should be avoided, if possible, by an appropriate early education and guidance of the patient. However, the clinician is often confronted with the delicate task to choose a reasonable balance between minimal and maximal diagnostic means that are available and treat the patient with respect, which is a basic requirement. A PET-CT Scan could have been a cost saving, sufficient alternative. According to Nahmias et al. (2007), it shows a specificity of 99% and a sensitivity of 48% in a preoperative lymph node staging of the neck with oral squamous cell carcinoma. A positive PET-CT scan has a high positive predictive value [21]. Although the low sensitivity doesn't really help the surgeon in the decision, whicht lymph node level has to be removed. Insofar, when planning a neck dissection, the surgeon shoud not depend on the PET-CT scan only. However, there are positive results in detecting remote metastases by PET-CT scans in other studies [22]. Schneider et al. (2006) report on the amenities of the PET-CT scan because of the exact anatomical representation in cases of the CUP-syndrome (cancer of unknown primary-syndrome) in order to find the primary tumor and in the diagnostic of a relapse in tumor aftercare [23]. All the authors mentioned above warn about false positive results, which can have drastic implications on the quality of life of the affected patient. PET-CT Scans are expensive and their use has to be limited to the proper indications. In the years to come, it will be a challenge for the professional medical associations to find consents that are consistent with both saving and optimizing the costs and taking into consideration the individual needs of the patients.

In conclusion, we point out that distant metastases of SCC's of the head and neck region can manifest themselves in the peripheral skeletal muscles and adjacent soft tissues. Therefore, during performing a tumor staging and during aftercare, this possible localization of metastases should be taken into consideration if suspect lesions were found in the peripheral skeletal muscles and nearby soft tissues in a patient who suffers from or who had once suffered from a SCC. It has to be considered that distant metastases of head and neck tumors are probably not the limiting factor for the patient's survival prognosis since the local primary tumors in the oral cavity can importantly impair nutrition and breathing. Therefore, an early operative treatment should be an option for patients suffering from a progressive and terminal head and neck tumor, as a considerable good life expectancy can generally result and as the quality of life can be improved or at least upheld on an acceptable level as long as possible.

## Competing interests

All authors have declared that no competing interests, whether of financial or non-financial nature exist.

## Authors' contributions

RS and OM designed the study. MBG and MH analyzed the data. RS, OM, MBG and MH contributed to writing the paper. DR supervised the clinical treatment and data collection. RS and OM wrote the main part of the paper. All authors gave useful comment on the analysis of data and text of the manuscript.

## Consent

Written informed consent was obtained from the patient for publication of this case report and accompanying images. A copy of the written consent is available for review by the Editor-in-Chief of this journal.
